# Unnecessary hospitalisations and polypharmacy practices in Tajikistan: a health system evaluation for strengthening primary healthcare

**DOI:** 10.1136/archdischild-2022-324991

**Published:** 2023-01-13

**Authors:** Sophie Jullien, Manzura Mirsaidova, Sitora Hotamova, Dilbar Huseynova, Gulnora Rasulova, Shoira Yusupova, Abdusamatzoda Zulfiya, Martin Weber, Susanne Carai

**Affiliations:** 1 Quality of Care and Patient Safety Office, World Health Organization, Regional Office for Europe, Athens, Greece; 2 Child and Adolescent Health, World Health Organization, Regional Office for Europe, Copenhagen, Denmark; 3 Ministry of Health and Social Protection of the Population, Dushanbe, Tajikistan; 4 City Health Department, Ministry of Health and Social Protection of the Population, Dushanbe, Tajikistan; 5 Pathology Department, National Centre for Obstetrics, Dushanbe, Tajikistan; 6 Tajikistan Country Office, World Health Organization, Dushanbe, Tajikistan; 7 Faculty of Health, School of Medicine, Witten Herdecke University, Witten, Germany

**Keywords:** Primary Health Care, Child Health, Child Health Services, Global Health

## Abstract

**Background:**

Children and pregnant women require multiple contacts with the healthcare system. While most conditions can be managed by primary healthcare (PHC) providers, hospitalisations are common. This health system evaluation in Tajikistan quantifies unnecessary and unnecessarily prolonged hospitalisations and assesses antibiotic and polypharmacy practices.

**Methods:**

Data were retrospectively collected from randomly selected medical records from 15 hospitals. Inclusion criteria were children 2–59 months of age with a primary diagnosis of acute respiratory infection or diarrhoea, or pregnant women with threatened preterm labour, threatened miscarriages, premature rupture of membranes or mild pre-eclampsia, hospitalised between January and September 2021.

**Results:**

Among 440 children and 422 pregnant women, unnecessary hospitalisations accounted for 40.5% and 69.2% of hospitalisations, respectively, ranging from 0% to 92.7% across the hospitals. Among necessary hospitalisations, 63.0% and 39.2% were unnecessarily prolonged in children and women, respectively.

Prior to admission, 36.8% of children had received antibiotics, in which more than half intramuscularly. During hospitalisation, 92.5% of children and 28.9% of women received antibiotics. Children and women received an average of 5 and 6.5 drugs, respectively; most were not indicated or with no evidence of benefits.

**Conclusions:**

The methodology is applicable across all health systems and can provide important insights on health service use and resource waste. Findings of this assessment in Tajikistan have led to evidence-based decisions and actions from stakeholders and policy makers with the goal of strengthening PHC and improving the management of common diseases in children and pregnant women.

**WHAT IS ALREADY KNOWN ON THIS TOPIC**

- Unnecessary hospitalisations and misuse of antibiotics cause harm and take a financial toll on users and health systems.
- Primary healthcare remains underused despite being key for achieving Universal Health Coverage.

**WHAT THIS STUDY ADDS**

- This health system evaluation in Tajikistan quantified the proportion of children and pregnant women hospitalised unnecessarily and for too long.
- It quantified misuse of antibiotics, including among children with diarrhoea, and the prescription of other medications with no evidence of benefits.

**HOW THIS STUDY MIGHT AFFECT RESEARCH, PRACTICE OR POLICY**

- Unnecessary and unnecessarily prolonged hospitalisations are a proxy for the underperformance of primary healthcare.
- The methodology can be implemented in other settings. Repeated at different points in time, it allows monitoring of health system performance.
- The Ministry of Health has committed to enquire the root causes of unnecessary hospitalisations and polypharmacy and to engage in a participatory policy dialogue.

## Background

Children under 5 years of age often get sick, and pregnant women require several antenatal care visits and may need medical care for pregnancy-related conditions. Most of these diseases and conditions can be entirely and safely managed at the primary healthcare (PHC) level. The global strategy of Integrated Management of Childhood Illness (IMCI) and the Integrated Management of Pregnancy and Childbirth (IMPAC) provide standards for the management of children and women at the PHC level and for the identification of those who need timely referral for hospital care.^1 2^

Observations from completed WHO assessment visits to countries in the WHO European Region showed that children with common conditions such as pneumonia or diarrhoea were often admitted to hospitals for treatment, but this was not quantified.^3^ Such conditions, however, could often be managed following the IMCI approach at the PHC level rather than in an inpatient context. Hospitalisation can lead to unnecessary psychological, emotional and physical disturbances and contribute to disruption of education, increased nosocomial infections and a larger financial burden incurred both by patients and health facilities.^4–10^ In addition, patients should be hospitalised only for the time that is strictly required.^11 12^

The Republic of Tajikistan is a landlocked country in Central Asia with 9 million inhabitants, of which a third are children aged 0–14 years.^13–15^ Respiratory infections and diarrhoeal diseases remain the most common causes of childhood hospitalisations and among the top causes of death.^16 17^ The fertility rate in the country (3.1 children per woman in 2021) is higher than globally and the other countries in Central Asia.^17 18^ Despite PHC being key for achieving Universal Health Coverage, especially in a mountainous country with added difficulties for transportation, PHC facilities remain largely underused.^19^ Data on unnecessary hospitalisation of children and pregnant women, that is, who could have been managed safely and entirely in a PHC setting, would provide a sound basis for discussion on the need to strengthen PHC and to improve the efficiency of health service delivery.

Previous observations in Tajikistan suggest that treatment of common childhood conditions often comprises multiple unnecessary and invasive drugs.^19^ Assessing the prescription of antimicrobials and other drugs in hospitalised children and women will help understanding the magnitude of the problem and allow targeted solutions.

This health system evaluation was conducted in Tajikistan with the aim to quantify unnecessary and unnecessarily prolonged hospitalisations in children and pregnant women and to quantify antibiotic use and polypharmacy in hospitalised children and pregnant women.

## Methods

### Study design

We conducted this health system evaluation in September–October 2021 in 15 public hospitals (online supplemental figure S1). WHO consultants and a technical working group established by the Ministry of Health and Social Protection of Population (MoHSPP), comprising obstetricians and paediatricians, travelled to the hospitals for data collection.

10.1136/archdischild-2022-324991.supp1 Supplementary data

**Figure 1:**
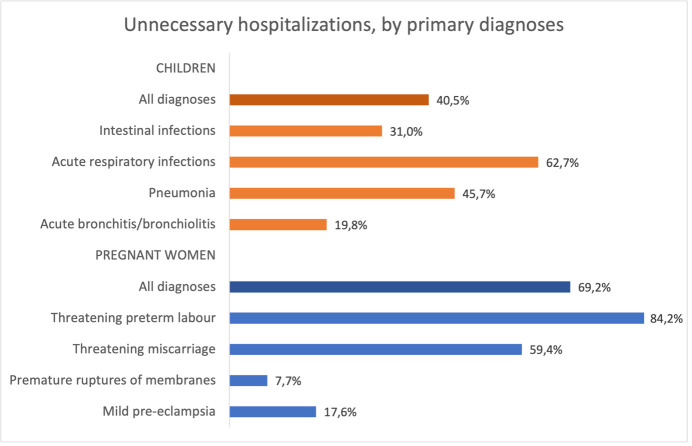
Proportion of unnecessary hospitalisations in children and pregnant women, among all diagnoses and by primary diagnoses.

### Inclusion criteria

We reviewed medical records of children 2–59 months of age hospitalised with a primary diagnosis of acute respiratory infection or intestinal infection (diarrhoea) and of pregnant women up to 37 gestation weeks hospitalised with a primary diagnosis of threatened preterm labour, threatened miscarriages, premature rupture of membranes or mild pre-eclampsia (table 1). These primary diagnoses are the most common causes of hospitalisations for these population groups in Tajikistan.^16^

**Table 1:** Inclusion criteria for evaluation of unnecessary hospitalisations

Population group	Inclusion criteria
Children	Hospitalised (admitted in the ward staying overnight). 2–59 months of age. Any of the following primary diagnosis (ICD-10^39^ code): Upper respiratory infection (J00-J06). Pneumonia (J12-J18). Acute bronchitis (J20). Acute bronchiolitis (J21). Other acute lower respiratory tract infection (J22). Intestinal infection (diarrhoea) (A00-A09).
Pregnant women	Hospitalised (admitted in the ward staying overnight). Confirmed pregnancy up to 37 gestation weeks. Any of the following primary diagnoses (ICD-10^39^ code): Threatened preterm labour up to 37 gestation weeks (O60).* Threatened miscarriages up to 22 gestation weeks (O20-O20.9). Premature rupture of membranes (O42.2). Mild preeclampsia (О14.0).

*Pregnant women with starting preterm labour with imminent preterm delivery are managed in the delivery department and were excluded from this evaluation.

ICD-10, International Statistical Classification of Diseases and Related Health Problems, 10th revision.

### Standard of care and determination of necessary or unnecessary hospitalisations

The reference for standard of care for children was the *WHO pocket book of Hospital care for children* as it is broadly used in Tajikistan and has already been used in similar assessments.^20 21^ For pregnant women, we used national protocols,^22–27^ endorsed by experts including from WHO and aligned with the IMPAC and the content of the effective perinatal care course that had been implemented in the country.^2^

For the classification of hospitalisation into necessary or unnecessary, we compared the clinical characteristics on admission from medical records against the standard of care (online supplemental tables S1 and S2). We classified hospitalisation as *necessary* if at least one criterion for hospitalisation was found, *unnecessary* if all the hospitalisation criteria were documented in the medical records and the child or woman did not meet any or *unclear* if information was missing. We classified hospitalisation as *unnecessarily prolonged* when the patient presented all discharge criteria for longer than 24 hours prior to discharge with no new hospitalisation criteria. All unnecessary hospitalisations were considered unnecessarily prolonged.

### Participant selection

In each hospital, the evaluation team randomly selected medical records (1 out of every 10 from the piles of medical records) from children and women hospitalised in January–September 2021, until obtaining 40 records for children and 40 for women. The number of 40 was chosen for feasibility and based on previous similar work.^21^

### Data collection, management and analysis

Data were extracted from medical records and recorded into an excel file together with the statement of necessary or unnecessary hospitalisation and prolonged hospitalisation. We analysed data with Microsoft Excel Program and Stata V.16.0.^28^ Comparison of proportions was performed using the χ^2^ test or Fisher’s exact test.

### National ethical clearance

Clearance from the MoHSPP was obtained in the context of the project for improving quality of hospital care, with Prikaz No 708 from 12 August 2021.

## Results

### Baseline characteristics

For children, 440 medical records met our inclusion criteria (online supplemental figure S2); their baseline characteristics are presented in table 2. The average age was 15.8 months (median 13, IQR 7–20.5). Overall, 205/440 (46.6%) children were referred from PHC or other hospitals, and 34/440 (7.7%) children were admitted at night (22:00–06:00). Children hospitalised with a primary diagnosis of a respiratory infection were classified in the medical records as pneumonia (94/440; 21.4%), acute bronchitis or bronchiolitis (86/440; 19.5%) and acute respiratory infection (118/440; 26.8%). The remaining 142 children (32.3%) had a primary diagnosis of diarrhoea or acute gastroenteritis, including one child with ascariasis and one child with dysentery and confirmed amoebiasis. Other diagnoses were common; the most commonly documented were anaemia (178/440; 40.5%) and ‘neurotoxicosis’ (52/440; 11.8%) (table 2).

**Figure 2:**
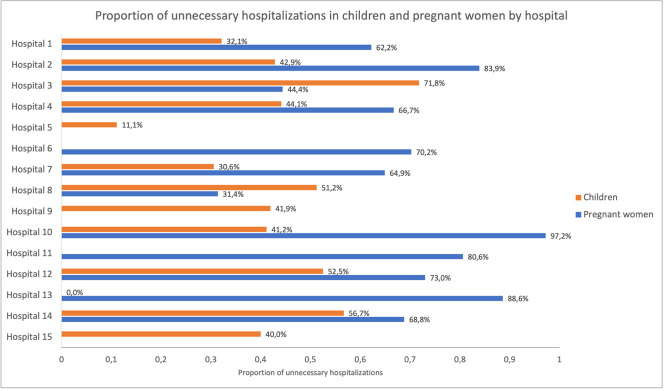
Proportion of unnecessary hospitalisations in children and pregnant women, by hospital.

**Table 2:** Baseline characteristics of children and pregnant women

	Children (n=440)	Pregnant women (n=422)
Age, median	13 months	24 years
Referral from PHC or other hospitals	205 (46.6%)*	273 (64.7%)
Night admission (22:00–06:00)	34 (7.7%)	33 (7.8%)
Primary diagnosis	Acute gastroenteritis: 142 (32.3%) Pneumonia: 94 (21.4%) Acute bronchitis or bronchiolitis: 86 (19.5%) Acute respiratory infection: 118 (26.8%)	Threatened preterm labour: 222 (52.6%) Threatened miscarriage: 170 (40.3%) Mild pre-eclampsia: 17 (4.0%) Premature rupture of membranes: 13 (3.1%)
Other diagnoses present at admission or during hospitalisation	Anaemia: 178 (40.5%) “Neurotoxicosis”†: 52 (11.8%) Malnutrition: 37 (8.4%) Perinatal encephalopathy: 24 (5.5%) Rickets: 11 (2.5%) Seizure: 10 (2.3%) Sepsis: 4 (0.9%) Cerebral palsy: 3 (0.7%)	Anaemia: 202 (47.9%) Chronic pyelonephritis: 92 (21.8%) Goitre: 12 (2.8%) Underweight: 9 (2.1%)

*Data collection on referral was missed for 30 children.

†’Neurotoxicosis’ is not recognised as a diagnosis according to the ICD-10 classification.

PHC, primary healthcare.

For pregnant women, 422 medical records met our inclusion criteria (online supplemental figure S3; table 2). The median age was 24 years. Two hundred and seventy-three of 422 (64.7%) women were referred from another health centre and 33/422 (7.8%) were admitted at night. Women were hospitalised due to threatened preterm labour (222/422; 52.6%), threatened miscarriage (170/422; 40.3%), mild pre-eclampsia (17/422; 4.0%) or premature rupture of membranes (13/422; 3.1%). Among other diagnoses, 202/422 (47.9%) pregnant women had anaemia and 92/422 (21.8%) had chronic pyelonephritis.

**Figure 3:**
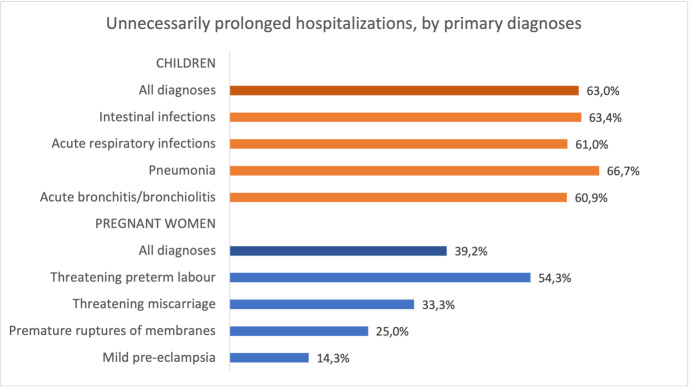
Proportion of unnecessarily prolonged hospitalisations among necessary hospitalisations in children and pregnant women, among all diagnoses and by primary diagnoses.

### Unnecessary hospitalisations

Overall, 178/440 (40.5%) children and 292/422 (69.2%) pregnant women were unnecessarily hospitalised. It was unclear whether hospitalisation was necessary in eight (1.8%) children (missing information in the medical records). The proportion of unnecessary hospitalisations varied between diagnoses both in children and women (7.7%–84.2%) (figure 1) and between hospitals (0%–71.8% for children and 31.4%–97.2% for women) (figure 2) but was similar between age groups (infants 2–11 months vs children 12–59 months), time of admission (day vs night), referral (referred from another health facility vs came by themselves) and between regional and city/district hospitals (online supplemental figures S4 and S5).

### Unnecessarily prolonged hospitalisations

The median duration of hospitalisation was 8 days (IQR 6–10) in children and 7 days (IQR 4–8) in women. The duration of hospitalisation did not differ between necessary and unnecessary hospitalisations. Differences by diagnoses and by hospitals are shown in online supplemental figures S6–S8.

Among necessary hospitalisations, 160/254 (63.0%) were unnecessarily prolonged in children (unclear for seven children) and 51/130 (39.2%) in women. The proportion of unnecessarily prolonged hospitalisations did not differ by type of diagnoses in children but ranged from 14.3% in women with mild pre-eclampsia to 54.3% in those with threatened preterm labour (figure 3) and varied by hospital (online supplemental figure S9).

Unnecessarily prolonged hospitalisations were more common among children of 12–59 months of age (69.3%) compared with infants (55.6%), although the difference was not statistically significant. Unnecessary prolonged hospitalisations were similar among type of hospitals and referral status (online supplemental figure S10).

### Antimicrobials

Prior to admission, 110/299 (36.8%) children received at least one antibiotic, corresponding to 77/194 (39.7%) children with a respiratory infection and 33/105 (31.4%) children with diarrhoea. These drugs were given intramuscularly in 64/110 (58.2%) children.

During hospitalisation, 407/440 (92.5%) children received at least one antimicrobial and 115/440 (26.1%) received at least two. Antibiotics were prescribed to 122/142 (85.9%) children with diarrhoea. Overall, the antibiotics most prescribed were ampicillin (received by 135/440 (30.7%) children), ceftriaxone (98/440; 22.3%), cefotaxime (45/440; 10.2%), gentamicin (34/440; 7.7%) and amikacin (29/440; 6.6%).

Overall, 122/422 (28,9%) women were prescribed antibiotics during hospitalisation, mostly ampicillin, ceftriaxone or metronidazole. The reason for prescription was frequently due to secondary diagnoses such as chronic pyelonephritis or respiratory infections, which were in most cases not confirmed clinically or by laboratory tests on admission but based on diagnoses reported in the referral paper and therefore likely to be unjustified.

### Polypharmacy

Hospitalised children and women received an average of 5 and 6.5 drugs, respectively. Drugs in children included oral rehydration salts, intravenous fluids and short-acting bronchodilators. Medications were often not indicated or with no evidence of benefits, such as antihistamines, probiotics, spasmolytics, mucolytics, interferon alfa-2b (Viferon), affinity-purified antibodies to human interferon gamma (Anaferon) and vitamin complex. Some children received intravenous insulin together with intravenous glucose solution.

Women were also commonly prescribed medications when they were not indicated or with no evidence of benefits, such as valerian, papaverine suppository, different types of vitamins, vitamin C and herbal tea for kidney diseases.

## Discussion

Unnecessary hospitalisations were common, accounting for 40.5% and 69.2% of hospitalisations in children and pregnant women, respectively. Among necessary hospitalisations, children and women were commonly kept too long when they could have safely been discharged. The misuse of antibiotics was considerable, which is of particular concern for the potential direct harm for the patients and for accelerating antimicrobial resistance, a global public health challenge.^29^ While antibiotics might well be indicated in children with severe pneumonia, the choice of antibiotics was not conformed to guidelines in a considerable proportion of cases. The situation is more worrying in children with diarrhoea, for which antibiotics are not indicated (except in case of dysentery) and can cause harm. Despite only one child with dysentery, 85.9% of children hospitalised with diarrhoea received antibiotics. In addition, children and women were commonly prescribed medication with no evidence of benefits.

Identifying contributing factors and understanding the rationale behind such practices (unnecessary hospitalisations and unnecessary medications) are primordial to allow targeted solutions. While quality education and training doctors on hospitalisation criteria and management of common diseases following standards of care are surely needed, other actions are also needed for improving quality of care.

National regulations that mandate hospitalisations probably contribute to unnecessary hospitalisations; for example, a referred patient coming at night needs to be hospitalised. In some cases, it is unclear whether practices are mandated by law or are the consequence of what healthcare worker believed to be regulated by law.

Allocation of public resources to the health sector is limited in Tajikistan, which is reflected in low salaries of health workers and high out-of-pocket payments for healthcare at all levels.^30^ Spending on medicines accounts for 37% of out-of-pocket payments.^31^ Reliance on informal payments to supplement salaries, and salaries and workforce directly linked to hospital bed occupancy, are likely to lead to non-evidence-based treatment and unnecessary hospitalisations. In addition, the common perception of patients and caregivers not feeling well taken care of if no medication is prescribed is likely to worsen the prescription of unnecessary drugs, despite the considerable cost incurred. Seven days’ treatment with four or five of the commonly used drugs was estimated, based on observations and data collected during this assessment, to a cost of 320 Tajikistani somoni (25 euros), which corresponds to around 20% of an average monthly salary. Investigations such as laboratory testing and parenteral medications are also expected by many patients to feel well taken care of. Awaiting investigations results and completing parenteral antibiotic courses (while sometimes not required or could have been given orally at home) could partly explained some of the prolonged hospitalisations. In addition, the Tajik population seems to consider hospital care superior to the care they could receive at PHC level. This surely lowers the threshold for admitting people to hospitals, leading to increased unnecessary hospitalisations. The PHC approach is key to achieving the goal of Health for All, which is physical, mental and social well-being for everyone in the community.^32–34^ Understanding community and health workers practices, perceptions and behaviours on healthcare and hospitalisation is key to success towards PHC and high-quality health services.^34^

This health system evaluation identified areas of concerns where qualitative research is warranted, including understanding the root causes of unnecessary hospitalisations.^35^ Based on these findings, the MoHSPP has committed to reviewing rules and regulations associated with hospitalisations, reviewing funding and financing systems for incentives for unnecessary hospitalisation, strengthening PHC and raising awareness on the harm of unnecessary hospitalisation and on the ineffectiveness of antibiotics for viral infections.

Another key achievement of this assessment is the identification of reliable indicators for monitoring progress. This health system evaluation in Tajikistan allowed to quantify children in hospitals that could have benefitted from PHC services. To the best of our knowledge, this kind of system evaluation has not previously been implemented elsewhere. The recognition of the need for reducing hospitalisations, however, is not new. A decade ago, researchers developed a tool to monitor potentially avoidable hospitalisations (those which might be avoided by government policies ensuring socioeconomic resources, access to timely, appropriate and affordable PHC, and the implementation of health promotion and disease prevention strategies) in children.^36 37^ By contrast with the potentially avoidable hospitalisations approach, which integrates many factors and is based on estimates, the methodology we used is easy to implement in all settings. Repeated at different points in time, the quantification of two specific and objective indicators (unnecessary hospitalisations and unnecessarily prolonged hospitalisations) allows monitoring the effectiveness of interventions for improving quality of hospital care. Such indicators may well have their role in measuring effectiveness, safety and indirectly access, within the health system performance assessment framework for universal health coverage.^38^

The limitations of this evaluation include the retrospective data collection from medical records. Information not recorded in the medical charts may lead to an overestimation of unnecessary and unnecessarily prolonged hospitalisations. For example hospitalisation of women or children due to social circumstances that might not be documented.

The strengths of this evaluation are multiple. Data were collected in 15 hospitals with different settings throughout Tajikistan (Soghd oblast, the Rayon Republican Subordinations and Dushanbe), making the findings most likely applicable to the whole country. Methodology was transparent, systematic and rigorous, making the evaluation reproducible for data comparison and tracking progress (monitoring) by following the same methodology, and for replication of this health system evaluation to other settings. This assessment focused on the most common diagnoses in hospitalised children and pregnant women in Tajikistan. The use of these findings for strengthening the management of these common diagnoses in PHC are likely to have a considerable impact in the overall quality of care for children and pregnant women.

## Conclusions

Findings of this assessment in Tajikistan have led to evidence-based decisions and actions from stakeholders and policy makers with the goal of strengthening PHC and improving the management of common diseases in children and pregnant women.

Unnecessary hospitalisations and unnecessarily prolonged hospitalisations constitute two objective indicators for evaluating and monitoring progress in health system performance to ensure universal health coverage.

## Data Availability

Data are available on reasonable request.

## References

[1] World Health Organization . Integrated management of childhood illness (IMCI). Geneva: World Health Organization, 2020.

[2] World Health Organization . Integrated management of pregnancy and childbirth: pregnancy, childbirth, postpartum and newborn care: a guide for essential practice. third edit. Geneva: World Health Organization, 2015. 26561684

[3] World Health Organization . European health information gateway. Available: https://gateway.euro.who.int/en [Accessed 6 Sep 2021].

[4] Goslin ER . Hospitalization as a life crisis for the preschool child. J Community Health 1978;3:321–46. 10.1007/BF01498508 730842

[5] Sheridan MS . Children’s Feelings About the Hospital. Soc Work Health Care 1975;1:65–70. 10.1300/J010v01n01_09 1235184

[6] Psychological RA . Emotional and physical experiences of hospitalized children. Clin Case Reports Rev 2016;2:10–13.

[7] Reyes LM , Khurana R , Liu F , et al . The impact of hospitalization on physical activity during pregnancy. J Obstet Gynaecol Can 2021;43:766–8. 10.1016/j.jogc.2020.09.018 34099221

[8] Heaman M . Psychosocial aspects of antepartum hospitalization. NAACOGS Clin Issu Perinat Womens Health Nurs 1990;1:333–41. 2206753

[9] Loos C , Julius L . The Client’s View of Hospitalization During Pregnancy. J Obstet Gynecol Neonatal Nurs 1989;18:52–6. 10.1111/j.1552-6909.1989.tb01617.x 2926523

[10] Organisation for Economic Co-operation and Development . Tackling wasteful spending on health highlights, 2017. Available: https://www.oecd.org/els/health-systems/Tackling-Wasteful-Spending-on-Health-Highlights-revised.pdf [Accessed 6 Sep 2021].

[11] Lehner DC , Sadler LS . Toddler developmental delays after extensive hospitalization: primary care practitioner guidelines. Pediatr Nurs 2015;41:236–42. 26665423

[12] World Health Organization Regional Office for Europe . Hospital care for children: quality assessment and improvement tool. Copenhagen: World Health Organization, 2015.

[13] The World Bank . Tajikistan - Population, 2020. Available: https://data.worldbank.org/indicator/SP.POP.TOTL?locations=TJ [Accessed 22 Feb 2022].

[14] Tajstat . Agency on statistics under president of the Republic of Tajikistan, 2020. Available: www.stat.tj [Accessed 22 Feb 2022].

[15] UNICEF . Country profiles - Tajikistan, 2022. Available: https://data.unicef.org/country/tjk/ [Accessed 22 Feb 2022].

[16] Republican Medical Statistics and Information Center of the Ministry of Health and Social Protection of the Population of the Republic of Tajikistan . Health care in the Republic of Tajikistan, 2021.

[17] Institute for Health Metrics and Evaluation . Tajikistan, 2022. Available: https://www.healthdata.org/tajikistan [Accessed 22 Feb 2022].

[18] United Nations Population Fund (UNFPA) . Tajikistan, 2021. Available: https://www.unfpa.org/data/demographic-dividend/Tj [Accessed 22 Feb 2022].

[19] World Health Organization . Regional office for Europe. assessment of sexual, reproductive, maternal, newborn, child and adolescent health in the context of universal health coverage in Tajikistan. Copenhagen. Licence: CC BY-NC_SA 3.0 IGO, 2021.

[20] World Health Organization . Pocket book of hospital care for children. guidelines for the management of common childhood illnesses. second edition. Geneva: World Health Organization, 2013. 24006557

[21] Lazzerini M , Shukurova V , Davletbaeva M , et al . Improving the quality of hospital care for children by supportive supervision: a cluster randomized trial, Kyrgyzstan. Bull World Health Organ 2017;95:397–407. 10.2471/BLT.16.176982 28603306 PMC5463809

[22] Ministry of Health and Social Protection of the Population of the Republic of Tajikistan . National standards: haemorrhages during pregnancy, delivery and postpartum, prevention, diagnosis and obstetric tactics, 2018.

[23] Ministry of Health and Social Protection of the Population of the Republic of Tajikistan . Clinical protocols on management of hypertensive disorders in obstetrics, 2015.

[24] Ministry of Health and Social Protection of the Population of the Republic of Tajikistan . Methodological recommendations: the procedures for organizing the hospital care for pregnant women, women in childbirth, postpartum and newborns in first, second levels of hospitals and perinatal centers, 2012.

[25] Ministry of Health and Social Protection of the Population of the Republic of Tajikistan . Methodological recommendations for development of local protocols: pathology conditions in pregnancy, 2012.

[26] Ministry of health and social protection of the population of the Republic of Tajikistan. National standards on management of high risk deliveries 2009.

[27] Ministry of health and social protection of the population of the Republic of Tajikistan. National standards on antenatal care 2008.

[28] StataCorp . Stata v.16. Texas, USA: College Station, 2019.

[29] World Health Organization . Antimicrobial resistance: global report on surveillance. Geneva: World Health Organization, 2014.

[30] Khodjamurodov G , Sodiqova D , Akkazieva B . Tajikistan: health system review. Health Syst Transit 2016;18:1–114. 27172509

[31] World Health Organization Regional Office for Europe . Health-Related SDG targets in Tajikistan: implementation of policies and measures for health and well-being. Copenhagen: World Health Organization, 2020.

[32] World Health Organization . Global strategy for health for all by the year 2000. World Health Forum 1981;2:1–90.

[33] International Conference on Primary Health Care . Declaration of Alma-Ata. Alma-Ata, USSR, 1978.

[34] Global Conference on Primary Health Care . Declaration of Astana. Astana, Kazakhstan, 2018.

[35] The Health Communication Capacity Collaborative (HC3) . Factors impacting the effectiveness of health care worker behaviour change.: a literature review. Baltimore: Johns Hopkins Center for Communication Programs, 2016.

[36] Anderson P , Craig E , Jackson G , et al . Developing a tool to monitor potentially avoidable and ambulatory care sensitive hospitalisations in New Zealand children. N Z Med J 2012;125:25–37. 23254524

[37] Craig E , Anderson P , Jackson G , et al . Measuring potentially avoidable and ambulatory care sensitive hospitalisations in New Zealand children using a newly developed tool. N Z Med J 2012;125:38–50. 23254525

[38] World Health Organization . Health system performance assessment. A framework for policy analysis. Papanicolas I, Rajan D, Karanikolos M, Soucat a, Figueras J, editors, 2022. 37023239

[39] World Health Organization . International statistical classification of diseases and related health problems 10th revision, 2016. Available: https://icd.who.int/browse10/2016/en [Accessed 18 Feb 2022].

