# Supplementary material for: Unnecessary hospitalisations and polypharmacy practices in Tajikistan: a health system evaluation for strengthening primary healthcare
Source: Arch Dis Child. 2023 Jan 13;108(7):531–7. doi: 10.1136/archdischild-2022-324991 (PMC10313957; doi:10.1136/archdischild-2022-324991)
Supplement: Supplementary data [file archdischild-2022-324991supp001.pdf]

# Unnecessary hospitalizations and polypharmacy practices in Tajikistan: a health system evaluation for strengthening primary health care

## Supplementary material

Sophie Jullien, Manzura Mirsaidova, Sitora Hotamova, Dilbar Huseynova, Gulnora Rasulova, Shoira Yusupova, Abdusamatzoda Zulfiya, Martin W. Weber, Susanne Carai.

### Figures

Figure S1. Cities where data were collected from 15 hospitals in Tajikistan. .......... 2

Figure S2. Flow diagram illustrating selection of medical records for children. .......... 3

Figure S3. Flow diagram illustrating selection of medical records for pregnant women. .......... 3

Figure S4. Proportion of unnecessary hospitalizations in children by age groups, time of admission, referral and hospital level. .......... 4

Figure S5. Proportion of unnecessary hospitalizations in pregnant women by time of admission and referral. .......... 4

Figure S6. Duration of hospitalization in children and pregnant women by primary diagnoses. .......... 5

Figure S7. Duration of hospitalization by hospital in children. .......... 5

Figure S8. Duration of hospitalization by hospital in pregnant women. .......... 6

Figure S9. Proportion of unnecessarily prolonged hospitalizations among necessary hospitalizations in children and pregnant women, by hospital. .......... 6

Figure S10. Proportion of unnecessarily prolonged hospitalizations among necessary hospitalizations in children, by age, referral and hospital level. .......... 7

### Tables

Table S1. Standards of care for assessment of unnecessary and unnecessarily prolonged hospitalizations in children .......... 8

Table S2. Standards of care for assessment of unnecessary and unnecessarily prolonged hospitalizations in pregnant women .......... 9

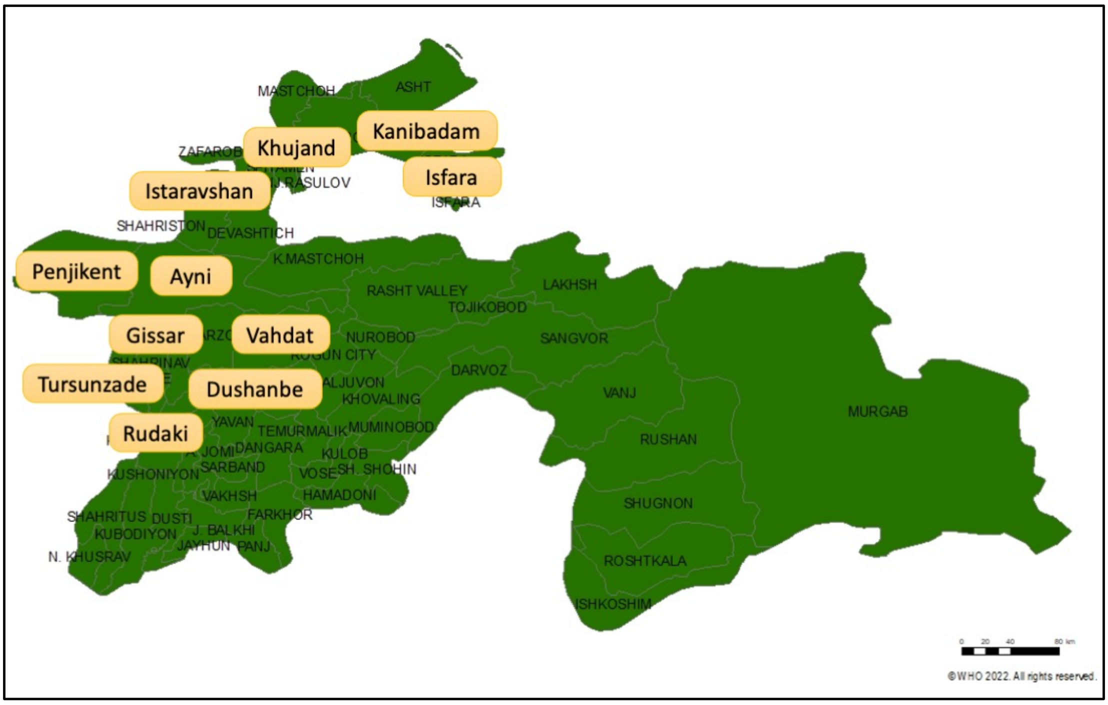


**Figure S1.** Cities where data were collected from 15 hospitals in Tajikistan.

These hospitals were selected by the Ministry of Health and Social Protection of Population and WHO for their involvement in the ongoing WHO project for improving the quality of hospital care, based on the low support received by development partners as compared to other regions in the country and their high-density catchment area.

**Regional hospitals**

1. Child Infection Hospital, Dushanbe (paediatric hospital, data collection for children only)
2. Istiqlol Hospital, Dushanbe (paediatric hospital, data collection for children only)
3. Republican Hospital Karabolo, Dushanbe
4. Khujand oblast child clinical hospital (paediatric hospital, data collection for children only)

**City/district hospitals**

5. Maternity #2, Dushanbe (maternity hospital, data collection for pregnant women only)

***Rayon Republican Subordinations***

6. Gissar Central District hospital
7. Rudaki Central District hospital
8. Tursunzade Central District hospital
9. Vahdat Central District hospital

***Soghd Oblast***

10. Ayni Central District hospital
11. Isfara Central District hospital
12. Istaravshan Central District hospital
13. Kanibadam Central District hospital
14. Khujand city maternity hospital (maternity hospital, data collection for pregnant women only)
15. Penjikent Central District hospital

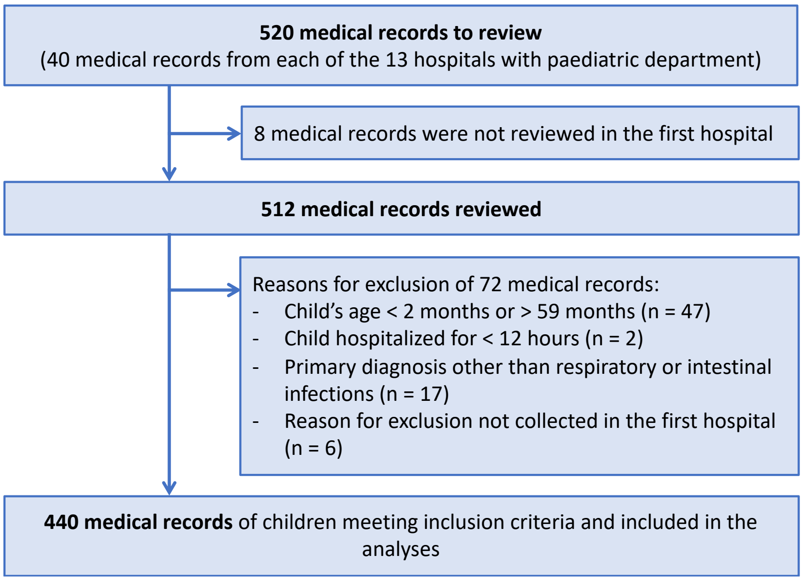


**Figure S2.** Flow diagram illustrating selection of medical records for children.

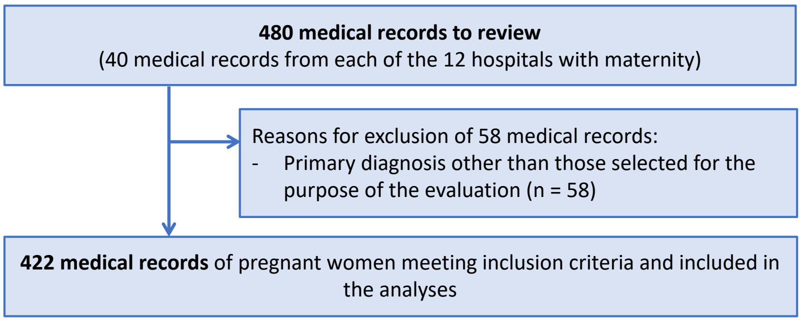


**Figure S3.** Flow diagram illustrating selection of medical records for pregnant women.



Jullien S, *et al. Arch Dis Child* 2023; 108:531–537. doi: 10.1136/archdischild-2022-324991

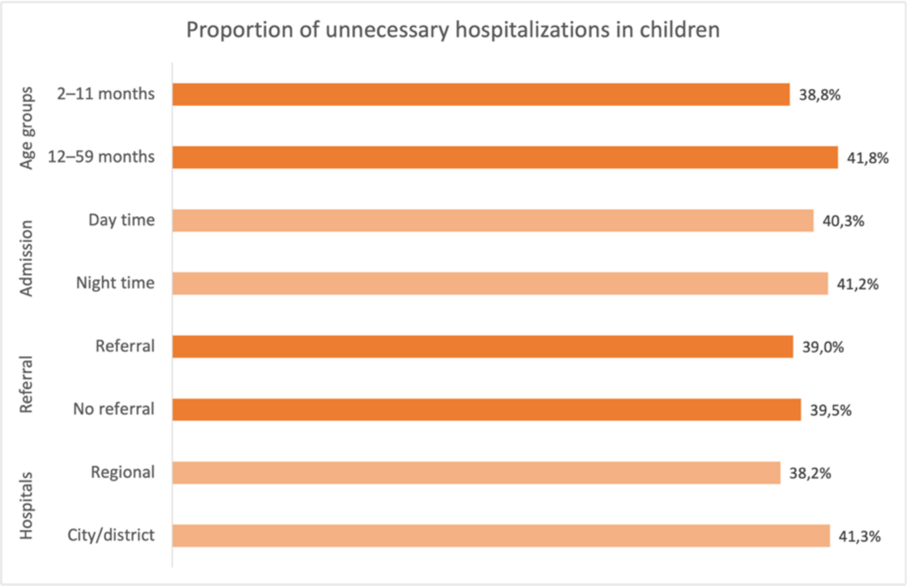


**Figure S4.** Proportion of unnecessary hospitalizations in children by age groups, time of admission, referral and hospital level.

*Note:* Comparison of proportions was performed using the chi-square test. For each group (age, admission, referral, hospitals), the difference of proportions was not statistically significant (p > 0.05).
Day time was defined as 6 am–10 pm.

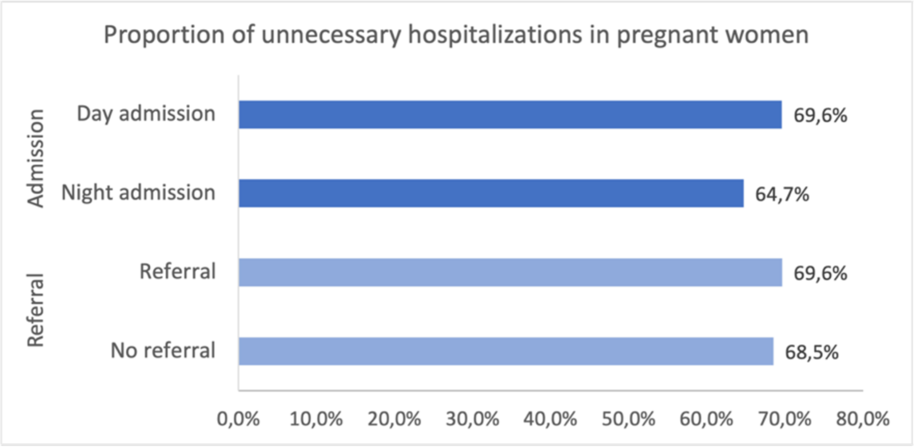


**Figure S5.** Proportion of unnecessary hospitalizations in pregnant women by time of admission and referral.

*Note:* Comparison of proportions was performed using the chi-square test. For each group (admission, referral), the difference of proportions was not statistically significant (p > 0.05).
Day time was defined as 6 am–10 pm.

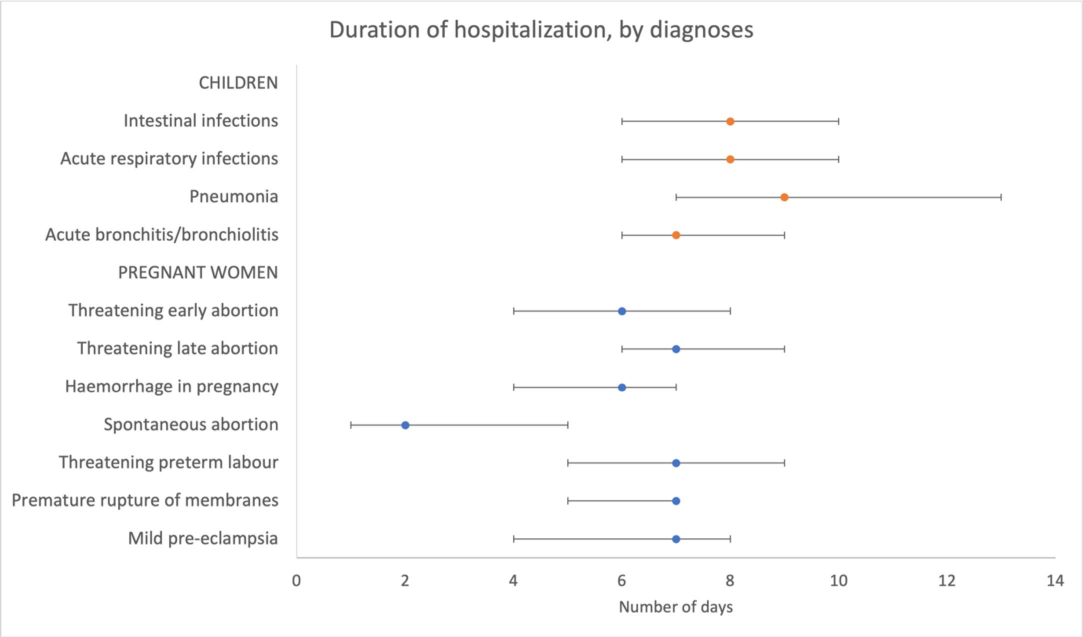


**Figure S6.** Duration of hospitalization in children and pregnant women by primary diagnoses.

The orange (children) and blue (pregnant women) dots show the median duration of hospitalization, in days, and the horizontal black line the interquartile range of duration of hospitalization, for each primary diagnosis. The primary diagnosis at admission of threatened miscarriage was disaggregated by diagnosis at discharge, as diagnoses at discharge are those directly related with duration of hospitalization: threatened early abortion (up to 12 weeks of gestation), threatened late abortion (13–22 weeks of gestation), haemorrhage in pregnancy, and spontaneous abortion.

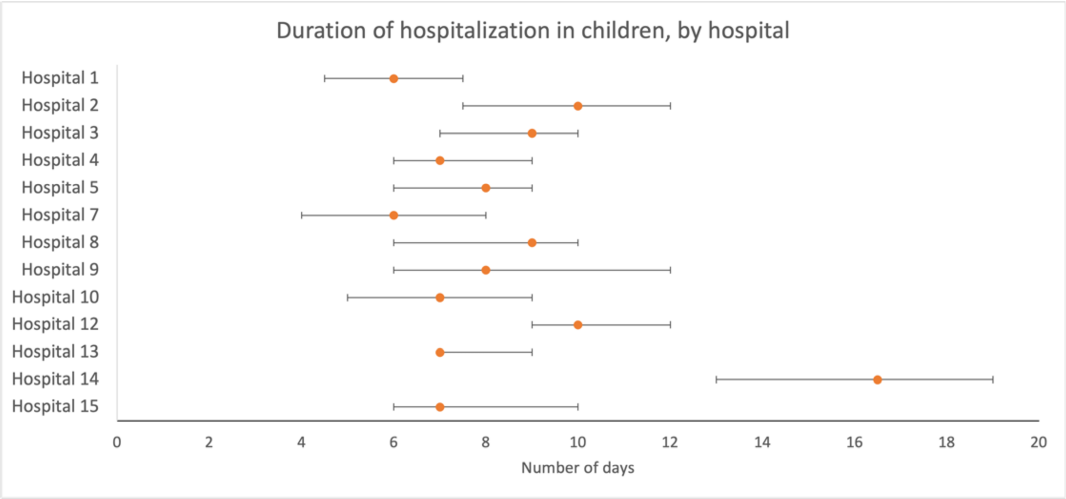


**Figure S7.** Duration of hospitalization by hospital in children.

The orange dots show the median duration of hospitalization, in days, and the horizontal black line the interquartile range of duration of hospitalization, for each hospital.

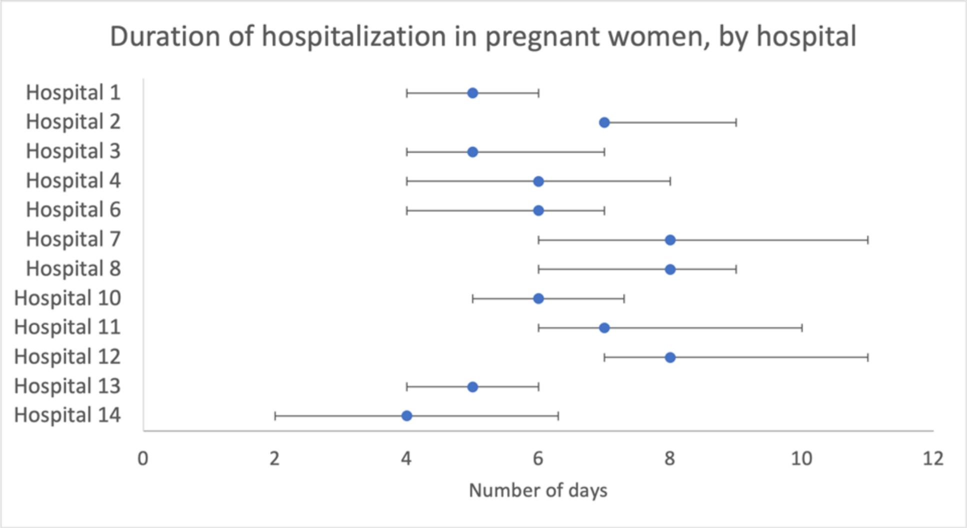


**Figure S8.** Duration of hospitalization by hospital in pregnant women.

The blue dots show the median duration of hospitalization, in days, and the horizontal black line the interquartile range of duration of hospitalization, for each hospital.

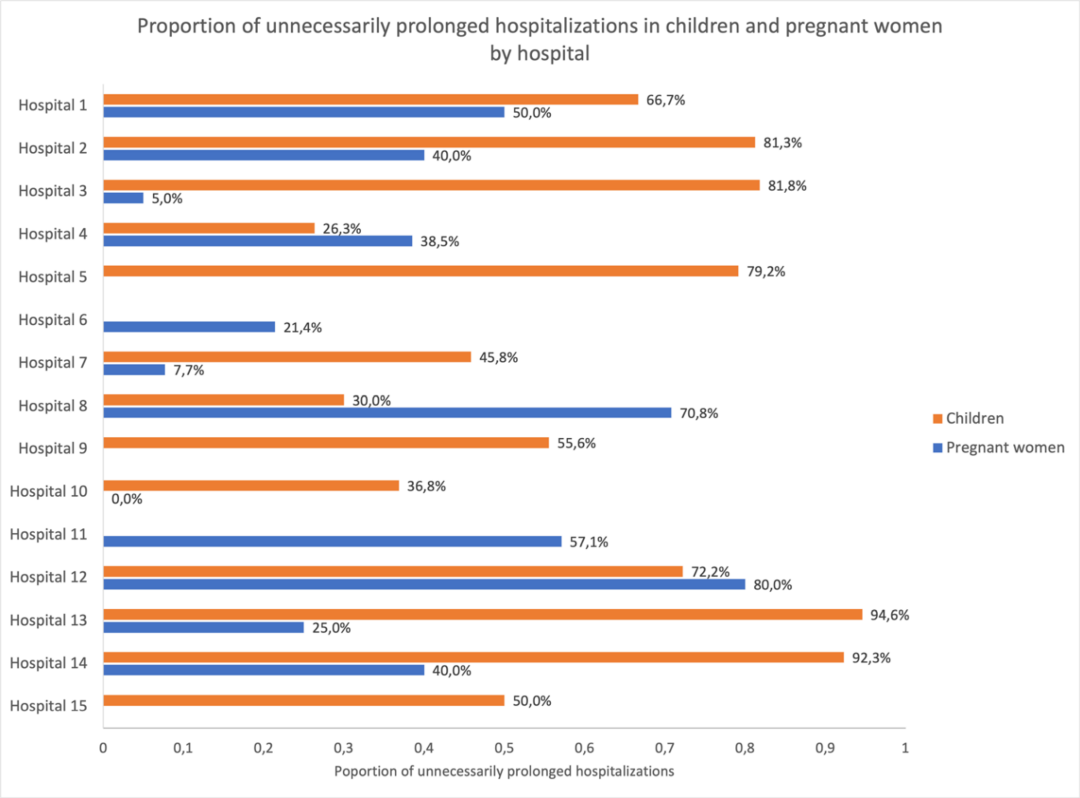


**Figure S9.** Proportion of unnecessarily prolonged hospitalizations among necessary hospitalizations in children and pregnant women, by hospital.

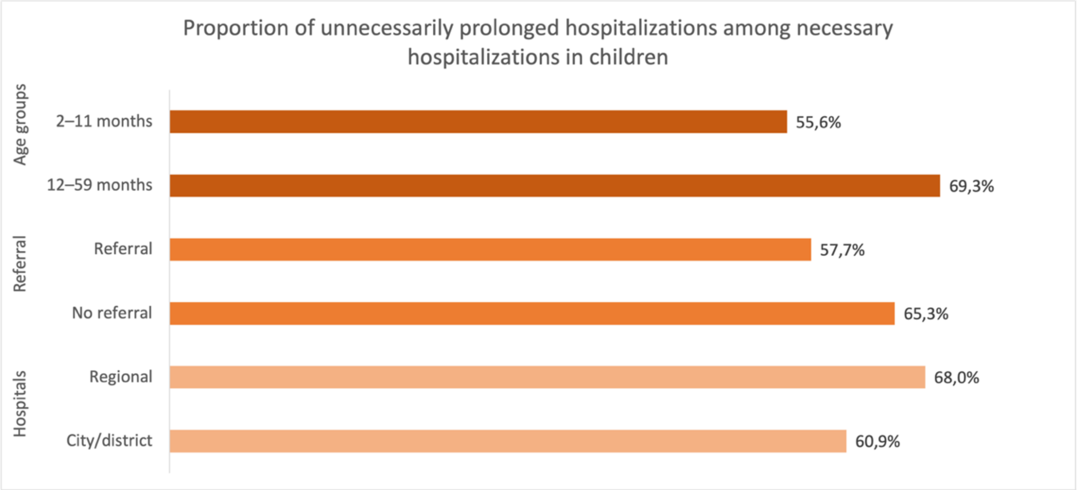


**Figure S10.** Proportion of unnecessarily prolonged hospitalizations among necessary hospitalizations in children, by age, referral and hospital level.

*Note:* Comparison of proportions was performed using the chi-square test or Fisher exact test. For each group (age, referral, hospitals), the difference of proportions was not statistically significant ($p > 0.05$).

**Table S1.** Standards of care for assessment of unnecessary and unnecessarily prolonged hospitalizations in children

| **Disease, condition** (ICD-10 code[1]) | **Hospitalization criteria[2]** | **Discharge criteria[2]** |
|---|---|---|
| **FOR ALL** | The presentation of any **general danger sign** entails a hospitalization criterion by itself:<br>• inability to drink or breastfeed,<br>• vomiting everything,<br>• history of convulsions during the current illness,<br>• lethargy, unconsciousness or convulsions. | A decision on when to discharge should be made on an individual basis, taking into consideration factors such as:<br>• the family's home circumstances and how much support is available to care for the child<br>• the staff's judgement of the likelihood that the treatment course will be completed at home or that the family will return immediately to hospital if the child's condition worsens. |
| **Upper respiratory tract infection including common cold and croup** (J00–J06) | Severe pneumonia (see "pneumonia" below)<br>Severe croup, defined by any of the following:<br>• Stridor even when the child is at rest<br>• Rapid breathing and low chest indrawing<br>• Oxygen saturation <90% or central cyanosis | • Respiratory distress resolved<br>• No hypoxaemia (SpO2>90%) on room air<br>• No apnoea<br>• No stridor<br>• Alert<br>• Afebrile<br>• No severe dehydration<br>• Feeding/eating well<br>• Not vomiting everything<br>• Able to take oral medication (if needed) |
| **Pneumonia** (J12–J18) | Severe pneumonia, defined by any of the following:<br>• Oxygen saturation <90% or central cyanosis<br>• Severe respiratory distress (grunting, very severe chest indrawing)<br>Pneumonia not improving after three days (of oral antibiotics) | |
| **Acute bronchitis** (J20)<br>**Acute bronchiolitis** (J21)<br>**Unspecified acute lower respiratory tract infection** (J22) | • Oxygen saturation <90% or central cyanosis<br>• Apnoea or history of apnoea<br>• Gasping and grunting (especially in young infants)<br>• Not improving 15 minutes after administration of rapid-acting bronchodilator (e.g. salbutamol)<br>- Signs of severe pneumonia (see above)<br>- Fast breathing: ≥ 50 breaths/minute in 2–11 months,<br>≥ 40 breaths/minute in 1–5 years | |
| **Diarrhoea, acute gastroenteritis, intestinal infectious diseases** (A00–A09) | Severe dehydration, defined as ≥ 2 of the following signs:<br>• lethargy or unconsciousness<br>• sunken eyes<br>• unable to drink or drinks poorly<br>• skin pinch goes back very slowly (≥ 2 sec) or "reduced turgor" | • No severe dehydration<br>• Feeding/eating well<br>• Not vomiting everything<br>• Alert<br>• Afebrile |
| | Severe persistent diarrhoea:<br>• diarrhoea lasting ≥ 14 days<br>• with signs of dehydration: see severe dehydration signs above, or ≥ 2 of the following signs: restlessness, irritability; sunken eyes; drinks eagerly, thirsty; skin pinch goes back slowly. | |
| | Dysentery (frequent loose stools mixed with blood) if any of the following criteria:<br>• < 2 months old<br>• severely ill children, who look lethargic, have abdominal distension and tenderness or convulsions | |

[1] ICD-10: International Statistical Classification of Diseases and Related Health Problems, 10th revision [20]
[2] Based on the WHO *pocket book of Hospital care for children* [21].

**Table S2.** Standards of care for assessment of unnecessary and unnecessarily prolonged hospitalizations in pregnant women

| **Disease, condition** (ICD-10 code[1]) | **Hospitalization criteria[2]** | **Discharge criteria[2]** |
|---|---|---|
| **Threatened preterm labour up to 37 gestation weeks** (O60) | Regular and irregular contraction of the uterus three or more in 30 minutes **WITH** at least one of the following:<br>• Rupture of foetal membranes<br>• Dilatation of the cervix of more than 2 cm<br>• Smoothing of the cervix of more than 80%<br>• Structural changes of the cervix<br>• Pain in the lower abdomen increasing in intensity | • No labour or contractions in 48 hours<br>• Full course of the respiratory distress syndrome prevention, received at least 48 hours |
| **Threatened miscarriages up to 22 gestation weeks** (O20–O20.9) | **ANY** of the following:<br>• Bloody discharge or bleeding<br>• Cramping pains in the lower abdomen<br>• Structural changes of cervix (the cervix skips the finger) | • No bleeding in 24 hours |
| **Premature rupture of membranes** (O42.2) | **ANY** of the following:<br>• Presence of amniotic fluid at instrumental examination with vaginal speculum<br>• Presence of fluid on the control pad within one hour<br>• Ultrasound examination suggestive of rupture of membranes | • Ultrasound test (amniotic fluid normal)<br>• Lack of amniotic fluid on control pad in 24 hours |
| **Mild preeclampsia** (O14.0) | • Blood pressure over 140/90 mmHg to 159/105 mmHg<br>**AND**<br>• Protein in urine 0.3 g/l to 3 g/l | • Blood pressure below 130/80 mmHg for two days<br>• Decreased urine protein below 0.2 g/l<br>• Gestation weeks ≤ 37 |

[1] ICD-10: International Statistical Classification of Diseases and Related Health Problems, 10th revision [20]
[2] Based on national protocols [23–28].
